# Preeclampsia Genomic Susceptibility Factors in Populations of African Ancestry: A Systematic Review and Meta-Analysis

**DOI:** 10.3390/ijms27062594

**Published:** 2026-03-12

**Authors:** Jonathan N. Katsukunya, Bianca Davidson, Khuthala Mnika, Nyarai D. Soko, Ayesha Osman, Mushi Matjila, Erika Jones, Collet Dandara

**Affiliations:** 1 Division of Human Genetics, Department of Pathology and Institute of Infectious Disease and Molecular Medicine (IDM), Faculty of Health Sciences, University of Cape Town, Cape Town 7700, South Africa; ktsjon001@myuct.ac.za (J.N.K.); k.mnika@uct.ac.za (K.M.); nyarai.soko@uct.ac.za (N.D.S.); 2 SAMRC/UCT Platform for Pharmacogenomics Research and Translation, South African Medical Research Council, Cape Town 7700, South Africa; bianca.davidson@uct.ac.za (B.D.); erika.jones@uct.ac.za (E.J.); 3 Division of Nephrology and Hypertension, Department of Medicine, Groote Schuur Hospital, Faculty of Health Sciences, University of Cape Town, Cape Town 7700, South Africa; 4 Kidney and Hypertension Research Unit, Faculty of Health Sciences, University of Cape Town, Cape Town 7700, South Africa; 5 Genetics of Inherited Kidney Diseases Africa (GIKD-Africa) Research Group, Division of Human Genetics, Department of Pathology, Faculty of Health Sciences, University of Cape Town, Cape Town 7700, South Africa; 6 Biomedical Sciences Unit, School of Allied Health Sciences, Harare Institute of Technology, Harare P.O. Box BE 277, Zimbabwe; 7 Department of Obstetrics and Gynaecology, Faculty of Health Sciences, University of Cape Town, Groote Schuur Hospital, Cape Town 7700, South Africa; ayesha.osman@uct.ac.za (A.O.); mushi.matjila@uct.ac.za (M.M.)

**Keywords:** preeclampsia, genomics, genetic susceptibility, epigenetics, Africa, Africans

## Abstract

The aim of this review is to examine the contribution of genomic variation to preeclampsia susceptibility in Africans. PubMed/Medline, Scopus, African Index Medicus and Sabinet African Journals databases were used to access studies conducted in populations of African descent focussing on the genomics of preeclampsia. Studies were selected according to PRISMA guidelines and assessed for quality and risk of bias using the Critical Appraisal Skills Programme (CASP) and Joanna Briggs Institute (JBI) checklists. Meta-analysis was conducted using a random effects model, and publication bias was evaluated using the Eggers test and funnel plots. Grading of Recommendations, Assessment, Development and Evaluation (GRADE) was applied to evaluate the certainty of evidence outcomes. Sixty-six (66) studies reporting on genomics of preeclampsia were retrieved. Forty-four (44) studies had a quality assessment score ≥75%. Vascular pathway genes (*GNB3*, *FLT1*, *NOS3* and *VEGFC*; OR (95% CI): 1.61 (1.38–1.88); *I*^2^: 0.0%, *p* = 0.87; GRADE: low certainty), immune/inflammatory pathway genes (*APOL1*, *ERAP2*, *HLA-G*, *IL-1β*, *LEPR* and *TNF-α*; OR (95% CI): 2.07 (1.68–2.54); *I*^2^: 42.2%, *p* = 0.04; GRADE: low certainty) and cellular homeostasis genes (*GLUT9*, *URAT1*, *SLC4A1* and *SLCO4C1*; OR (95% CI): 1.65 (1.43–1.91); *I*^2^: 0.0%, *p* = 0.99; GRADE: low certainty) showed pooled effect estimates suggestive of moderate to increased preeclampsia risk. *APOL1* G1 or G2 risk alleles seemed to contribute 1.70-fold (95% CI: 1.39–2.07; *I*^2^: 0.0%; *p* = 0.51; GRADE: low certainty), respectively, to overall preeclampsia risk. Vascular, immune/inflammatory and cellular homeostasis genes may be ideal starting points for future research, and further validation of the role of *APOL1* G1 or G2 risk alleles in preeclampsia may be essential.

## 1. Introduction

Preeclampsia is among the leading contributors to pregnancy-related deaths, accounting for an annual death toll of 76,000 and 500,000 women and children, respectively [1,2,3]. These deaths appear to be disproportionately higher in developing countries within the Sub-Saharan African region, as it has the highest reported prevalence of preeclampsia to date [2,4]. The prevalence of preeclampsia within the Sub-Saharan region has been reported to be as high as 16.7% [4,5] and intrinsic and extrinsic factors have been shown to contribute to this. However, evidence suggests a strong genetic involvement, with African women bearing a significant burden compared to non-African women [4].

The International Society for the Study of Hypertension in Pregnancy (ISSHP) defines preeclampsia as new onset hypertension (blood pressure (BP) ≥ 140/90 mmHg) occurring from the 20th week of pregnancy, accompanied by either any of or the combination of the following: (i) proteinuria (≥30 mg/mmol urinary protein), (ii) other maternal end-organ dysfunction (i.e., neurological complication, pulmonary oedema or acute kidney injury (AKI)), and (iii) evidence of utero-placental dysfunction (i.e., placental abruption, angiogenic imbalance or foetal growth restriction) [6].

The exact cause of preeclampsia remains unknown, and it has been termed a disease of theories since the 1900s [7]. Evidence to date suggests that abnormal placentation and decreased trophoblastic invasion are a major cause in its early-onset phenotype. This leads to poor utero-placental perfusion, hypoxia, oxidative stress and a sustained release of anti-angiogenic factors and inflammatory mediators (triggering chronic inflammation) into the bloodstream. Ultimately, widespread endothelial dysfunction occurs, causing vasoconstriction and end-organ damage [8], which also partly explains the occurrence of adverse outcomes such as kidney damage or subsequent cardiovascular diseases that may develop long-term. Evidence also suggests that environmental factors have a significant role to play, and they have been shown to interfere with trophoblast invasion and placentation [9].

These lines of evidence provide a sufficient rationale for the involvement of genetic and even epigenetic factors in the aetiology of preeclampsia, especially considering that the underlying pathways are regulated by many genes and that environmental stressors often lead to DNA modifications (i.e., histone modifications, acetylation or methylation). DNA methylation seems to be widely reported in preeclampsia, and differential methylation patterns in several genes (i.e., *FLT1*, *KOR1*, *MTHFR* and *VEGFA*) have been shown to play a role in preeclampsia susceptibility [10].

This causes us to question whether genetic or epigenetic factors have utility in preeclampsia detection. Preeclampsia is manageable, especially when it is detected early. According to the ISSHP, early identification of individuals at high-risk is currently based on suggestive clinical risk factors (i.e., medical history, pre-existing hypertension, comorbidities, biochemical markers and/or ultrasonography) and some prognostic tools have been developed to this effect, such as the Foetal Medicine Foundation web-based tool [9,11,12]. However, current strategies and tools still lack specificity and there are reports that they fall short on their ability to resolve all cases, highlighting a need for improved strategies considering an individual’s personalised risk. It can be argued that the lack of some preeclampsia risk factors could contribute to this, such as genetic and possibly epigenetic markers, and this may be limiting the predictive or prognostic ability of current tools. This is a case in point for women with no history of preeclampsia or presenting with no suggestive clinical risk factors.

The Fourth Industrial Revolution (4IR) has fostered key technological advancements and artificial intelligence (AI) lies at the core of these advancements. The ‘AI boom’ in the 2010s catapulted the transition from traditional statistical modelling in preeclampsia to the utilisation of machine learning (ML) approaches, which have been demonstrated to have a higher preeclampsia prediction yield [13], overcoming most traditional statistical modelling approaches. The increase in computing power and subsequent drops in computing costs has further spurred technological advancements in the 4IR post 2020 (also referred to as the ‘AI spring’), leading to the development of sophisticated AI-based models (i.e., deep learning (DL) models) capable of handling complex datasets and even robust prediction [14]. These have now started to gain prominence and may represent the next frontier of precision medicine.

Within the context of preeclampsia, efforts are ongoing on the utilisation of these 4IR technologies in preeclampsia prediction [13], but it seems that understanding of genetic or epigenetic markers remains sparse, hindering subsequent incorporation in ML-based predictive tools [15], especially for high-risk African populations. Given the modest body of work on the genomics of preeclampsia in African populations and the emerging proliferation of 4IR technologies, now is an opportune time to conduct a systematic review and meta-analysis to quantify pooled genomic susceptibility in Africans, particularly in pathways known to be involved in its pathology. In this review, we aim to evaluate the association of genetic and epigenetic determinants of preeclampsia susceptibility in African populations. We propose a theoretical framework, highlighting potential places where 4IR technologies and roles of genetic or epigenetic factors can be utilised in early detection and subsequent management of preeclampsia and long-term health outcomes.

## 2. Material and Methods

### 2.1. Search Strategy

A literature search was conducted by accessing the PubMed/Medline, Scopus, African Index Medicus (AIM) and Sabinet African Journals databases for studies published up to and including 31 December 2025. The search was limited to full length articles written in English. To tease out studies for inclusion in this review, a combination of Medical Subject Headings (MeSH) (Supplementary Methods) were used. This review was conducted following the systematic review methodology to ensure a comprehensive search and high-quality reporting; the protocol was registered on PROSPERO (CRD420261297255).

### 2.2. Eligibility Criteria

The PICO (Population, Intervention/Exposure, Comparison and Outcome(s)) framework, with the following elements: (i) Population: African women who develop preeclampsia during pregnancy or non-pregnant women with a history of preeclampsia during pregnancy; (ii) Intervention: exposure to any genetic or epigenetic factors (i.e., mutations, single nucleotide polymorphisms (SNPs), haplotypes or DNA methylation patterns or any epigenetic change (acetylation, histone modification or chromatin remodelling)) to preeclampsia; (iii) Comparison/comparator: African normotensive women or women who do not develop hypertension during pregnancy or preeclampsia; and (iv) Outcome: development or occurrence of preeclampsia and/or subtypes. This led to the development of the following review question, “What is the contribution of predisposing genetic or epigenetic factors to susceptibility to preeclampsia in African women?”.

### 2.3. Study Selection and Screening

**Figure 1:**
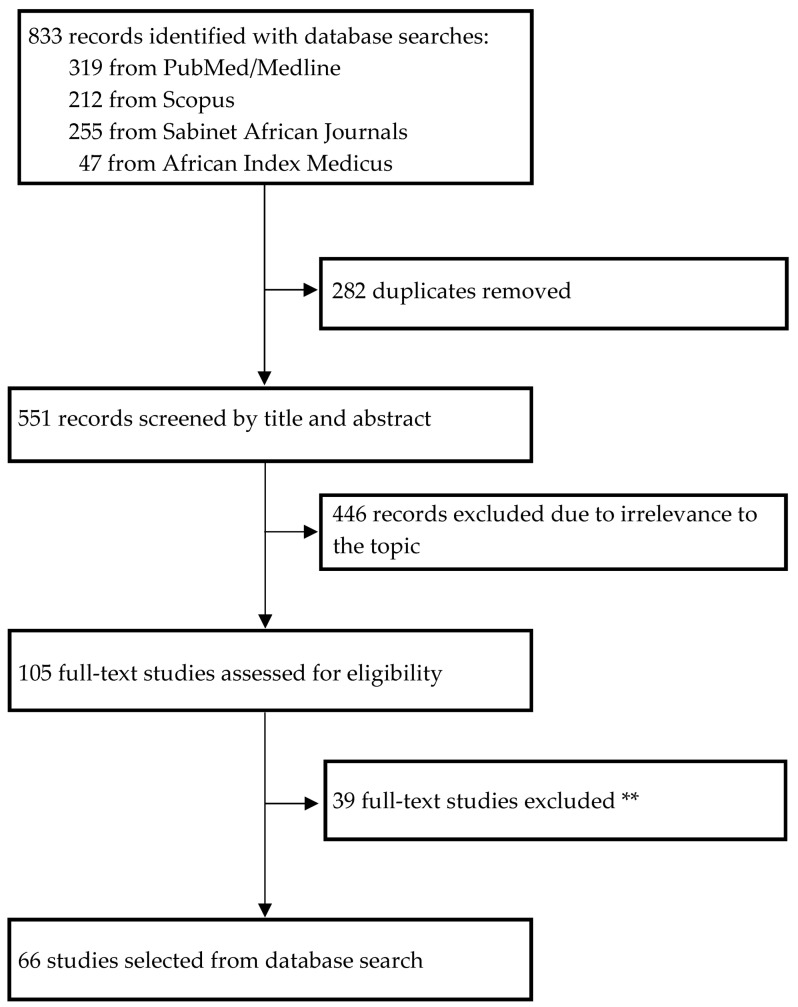
Figure diagram of study selection according to the Preferred Reporting Items for Systematic reviews and Meta-analyses (PRISMA) 2020 guidelines. ** The search outputs from the four databases were compared to remove duplicates. Initial screening step of the remaining articles achieved through assessment of the titles and abstracts to check if they satisfy the inclusion criteria. Second screening step involved assessment of article eligibility, and eligible studies were population-based studies (i.e., retrospective or prospective cohort or case–control studies), clinical trials, case reports, and published controlled studies. Narrative or systematic reviews, study protocols, short communications and conference proceedings were excluded.

Studies satisfying the following inclusion criteria: (i) genetic and epigenetic studies conducted in individuals of African descent (i.e., Africans (including those of Mixed Ancestry descent), African Americans, and Afro-Caribbeans); (ii) genetic and epigenetic studies whose main outcome of interest was defined as preeclampsia and/or subtypes (early onset preeclampsia (EOPE), late onset preeclampsia (LOPE), eclampsia or severe preeclampsia (i.e., HELLP (Haemolysis, Elevated Liver enzymes and Low Platelets) syndrome, BP ≥ 160/110 mmHg; maternal neurological disorders such as persistent headaches and brisk reflexes, eclampsia, acute pulmonary oedema, proteinuria ≥ 5 g/day, oliguria < 500 mL/day, creatinine > 120 μmol/L, thrombocytopenia < 100,000/mm^3^, intrauterine growth restriction, oligohydramnios, or foetal death in utero); and (iii) studies with sufficient genetic or epigenetic data, including odds ratios/risk ratios (ORs/RRs) and confidence intervals (CIs), were considered eligible. The exclusion criteria were (i) genetic or epigenetic studies for gestational hypertension only or hypertension in pregnancy only (i.e., preeclampsia not explicitly stated or defined) and (ii) genetic or epigenetic studies done in non-African populations (i.e., European or Asian). All authors independently screened the titles and abstracts and assessed the full texts of all potentially eligible studies (Figure 1).

### 2.4. Data Extraction

Data from the retrieved studies were extracted independently by three investigators (JK, EJ and CD). The data extracted from each study reporting on genetics or epigenetics of preeclampsia included: (i) gene(s) studied, (ii) pathway or function affected, (iii) SNPs or mutations investigated, (iv) population group, (v) sample size (N), (vi) main findings and (vii) effect measures (ORs/RRs and CIs). These are subdivided into studies reporting on maternal genetics and studies reporting on maternal and foetal genetic interactions on susceptibility to preeclampsia and/or its subtypes.

### 2.5. Study Quality Assessment

Quality assessment or critical appraisal of eligible studies was conducted (by JNK, NS and KM) and validated (by BD, AO, MM, EJ and CD) using the Critical Appraisal Skills Programme (CASP) checklists for case–control/cohort studies; meanwhile, the Joanna Briggs Institute (JBI) checklist for case reports was used for any retrieved case reports. Each question on the checklist was given a total weighting of 5 in each checklist, where 0 is very low and 5 is very high. The total score for each study was expressed as a percentage (%) and studies with scores ≥ 75% indicated high quality or rigour, with low risk of bias. If the scores were between 50 and 74%, the studies were considered to be of average quality, while studies with total scores < 50% were considered to have high-risk of bias.

### 2.6. Statistical Analyses

We conducted a meta-analysis using the ‘meta’ package in R studio (v4.4.1), specifically, the ‘metagen’ function for generic inverse-variance meta-analysis, which takes as input each study’s treatment effect (i.e., log ORs in this case) and the standard error of the treatment effect (i.e., log upper 95% CI–lower 95% CI/2 × 1.96). Based on domain knowledge, the random effects model was assumed because heterogeneity of the included studies was obvious (i.e., resulting from different SNPs studied in different genes). As a result, the DerSimonian–Laird random effects model was used to combine the effect of each SNP across studies yielding overall pooled ORs. African populations are a genetically heterogenous group and have been reported to harbour the greatest depth of genetic variation. However, for this meta-analysis, we treated all individuals of African descent as one unit, with the goal of exploring how each SNP in each pathway collectively contributes to susceptibility to preeclampsia.

Therefore, as exploratory analysis, the combined pooled effects of SNPs specific to each biological pathway or function (where possible) were determined. If there were ≥3 studies reporting on a particular SNP, the pooled effect in that single gene was determined. Sensitivity analyses were performed using the leave-one-out method to demonstrate the robustness of the study findings. Forest plots visualised the individual SNP effect sizes, the pooled effect sizes and heterogeneity across studies. The *I*^2^ values indicated the extent of heterogeneity and describe the percentage (%) of total variation across studies that is a result of heterogeneity, not sampling error [16]. *I*^2^ ≤ 50% was suggestive of low to moderate heterogeneity and *I*^2^ > 50% indicated considerable to high heterogeneity. Publication bias was assessed visually using funnel plots and statistically using the Eggers test. *p* < 0.05 for the Eggers test indicated potential publication bias. Trim-and-fill analysis (where applicable) was then conducted to explore the potential effect of publication bias.

### 2.7. Assessment of Certainty of Evidence

The Grading of Recommendations, Assessment, Development and Evaluation (GRADE) tool was applied to ascertain the certainty of evidence obtained for each pooled effect estimate. Briefly, the GRADE tool allows for evaluation of the quality of evidence taking into account study design, risk of bias, inconsistency (or heterogeneity), indirectness, imprecision and publication bias [17].

## 3. Results

### 3.1. Search Results and Study Characteristics

A total of 833 (PubMed/Medline: 319, Scopus: 212; AIM: 47; Sabinet African Journals: 255) studies were retrieved using our search terms. After removal of duplicates and irrelevant studies, 105 studies remained. These studies were further assessed for eligibility through full-text screening, and 39 studies did not satisfy the inclusion criteria. Therefore, 66 full-text studies were retained. Overall, 25% of the retrieved studies were conducted in African American populations and 75% in continental Africans, all reporting on maternal and/or foetal genetics or epigenetics on susceptibility to preeclampsia (Supplementary Table S1 and Table 1).

**Figure 2:**
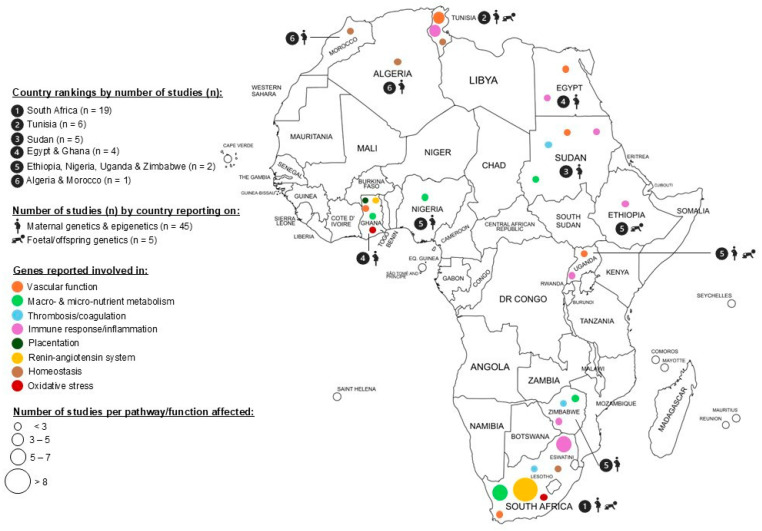
Total number (*n*) of continental African genomic and epigenomic studies reporting on association. maternal or foetal/offspring genetic variation with susceptibility to preeclampsia and/or its subtypes. The contribution of foetal genetic variation included in studies from Ethiopia (*n* = 2), Uganda (*n* = 1), Tunisia (*n* = 1) and South Africa (*n* = 1) only. The contribution of epigenetics (i.e., DNA methylation) included in studies from Nigeria only (*n* = 1).

The functional significance of SNPs most significant in African populations is shown in Table 2. Figure 2 maps the retrieved studies done in continental Africans. These studies are represented by South African, Tunisian, Sudanese, Egyptian, Ghanaian, Zimbabwean, Nigerian, Ugandan, Moroccan and Algerian population groups. Most studies adopted a case–control study design, except one cohort study [30] and one case report [31].

### 3.2. Quality of Eligible Studies, Effect Sizes and Certainty of Evidence

**Figure 3:**
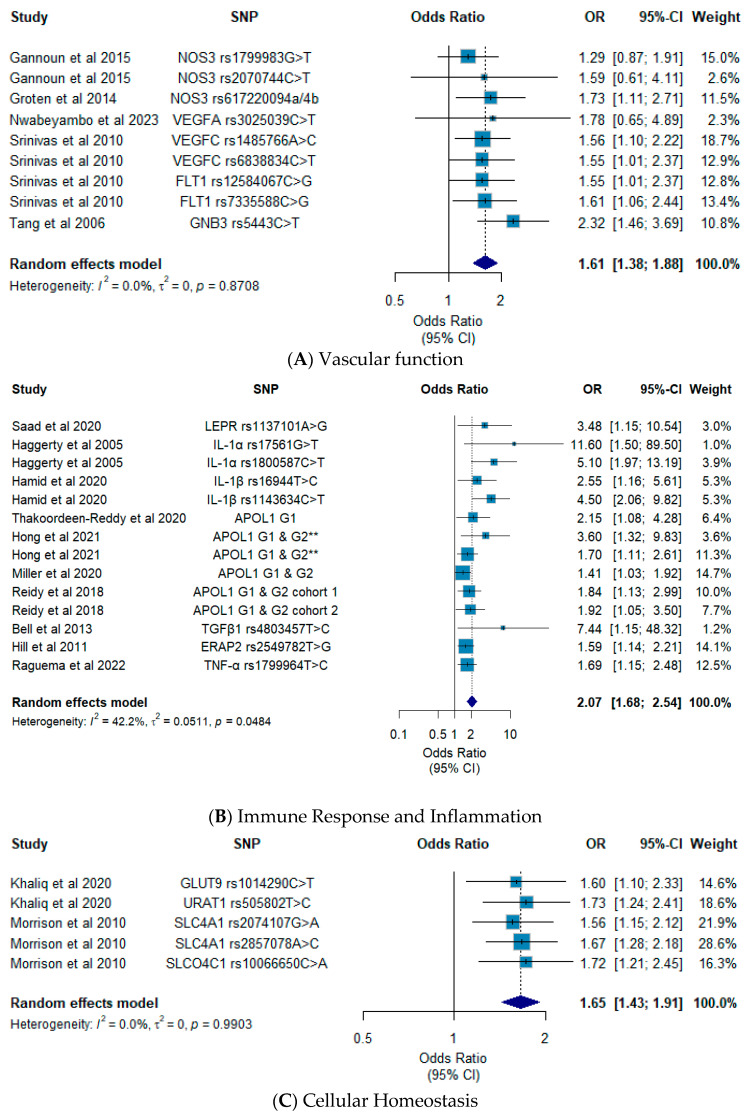
Exploratory meta-analyses pooling SNPs affecting vascular function (**A**), immune response and inflammation (**B**) and cellular homeostasis (**C**). ** denotes association under the recessive and additive models of inheritance. Refs. [19,21,22,23,24,29,31,32,33,34,35,36,37,38,39,40,41,42,43].

**Figure 4:**
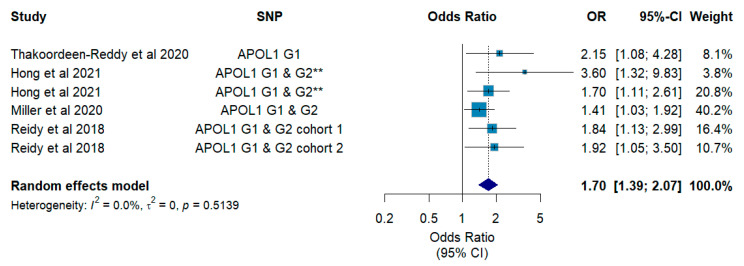
Forest plot indicating the magnitude of effect of SNPs reported for *APOL1*. (Pooled effect of SNPs reported in *APOL1*). ** denotes association under the recessive and additive models of inheritance. Refs. [21,23,24,40].

Scores from the quality assessment of each eligible study are presented in Supplementary Tables S2–S4. Thirty-two (32) studies scored ≥ 75%, 26 studies scored between 50 and 74% and 8 studies scored < 50%. Since most studies were observational (i.e., case–control) by design, the certainty of evidence was low (Supplementary Table S5) according to the GRADE tool. When genes belonging to the same pathway or affecting the same biological function were pooled as exploratory, it appears that genetic dysregulation in (i) vascular function (*GNB3*, *FLT1*, *NOS3*, *UTS2* and *VEGFC*; (OR (95% CI): 1.61 (1.38–1.88); *I*^2^: 0.0%, *p* = 0.87; GRADE: low certainty), (ii) immune response/inflammation (*APOL1*, *ERAP2*, *HLA-G*, *IL-1β*, *LEPR* and *TNF-α*; OR (95% CI): 2.07 (1.68–2.54); *I*^2^: 42.2%, *p* = 0.04; GRADE: low certainty) and cellular homeostasis (*SLC4A1*, *SLCO4C1*, *GLUT9* and *URAT1*; OR (95% CI): 1.65 (1.43–1.91); *I*^2^: 0.0%, *p* = 0.99; GRADE: low certainty) significantly to susceptibility to preeclampsia. Per gene/SNP pooling was possible for *APOL1* G1 or G2 risk alleles and showed that the combined effect on overall preeclampsia susceptibility was nearly 2-fold (OR (95% CI): 1.70 (1.39–2.07); *I*^2^: 0.0%, *p* = 0.51; GRADE: low certainty) (Figure 3 and Figure 4). Sensitivity analyses are shown in Supplementary Tables S6–S9, and no study was found to have a considerable effect on the pooled estimates.

### 3.3. Publication Bias Assessment

Funnel plots (Supplementary Figure S1 and S2) seemed asymmetrical from visual inspection, indicating potential publication bias, except for SNPs affecting cellular homeostasis (Supplementary Figure S1B). The Eggers test assumptions were valid for SNPs affecting immune response/inflammation (*I*^2^ = 42.2%, *n =* 14) at *p* < 0.0001, confirming potential publication bias. Although significant publication bias was observed for SNPs affecting immune response/inflammation, trim-and-fill analysis imputed six additional studies, suggestive of potential small study effects. However, the adjusted pooled effect for SNPs affecting immune response/inflammation still pointed towards increased risk of preeclampsia (OR (95% CI): 1.73 (1.31–2.28)) (Supplementary Figure S2).

## 4. Narrative Synthesis

### 4.1. Role of GNB3, FLT1, NOS3, UTS2 and VEGFC in Vascular Function and Susceptibility to Preeclampsia

*FLT1* (also known as VEGFR-1) located on chromosome 13q12.3 encodes for an fms-related tyrosine kinase 1, a member of the vascular endothelial growth factor receptor (VEGFR) family. *FLT1* has 32 exons and several transcript variants encoding different isoforms have been reported, including full-length transmembrane receptor isoforms and shortened, soluble isoforms. Soluble isoforms are the ones mostly implicated in the pathogenesis of preeclampsia, and polymorphisms such as rs12584067G>C (c.3287-523G>C) and rs7335588C>G (c.1437-4471C>G) have been found to be associated with susceptibility to preeclampsia in Africans. A study by Srinivas et al., (2010) found rs12584067G and rs7335588G alleles to be associated with nearly double the risk (Figure 3A) of developing preeclampsia in African American women [32]. It is important to note that *FLT1* has been validated for its functional role in preeclampsia and a recent study reported it to be among the highly upregulated genes in placentas isolated from African women with severe preeclampsia [44].

FLT1 binds to several vascular endothelial growth factors such as VEGFA, VEGFB, VEGFC encoded for by *VEGFA* (chromosome 6p21.1), *VEGFB* (chromosome 11q13.1) and *VEGFC* (chromosome 4q34.3) respectively, all of which have been reported to play a role in preeclampsia. *VEGFA* and *VEGFC* are widely reported in Africans (Supplementary Table S1), but *VEGFC* rs1485766A>C (c.705-1803A>C) and rs6838834C>T (c.148-2698G>A) SNPs, have been shown to be associated with moderate to increased risk (Figure 3A) of preeclampsia. Particularly, African American women carrying the rs1485766A and rs6838834C alleles were at a significantly elevated risk of preeclampsia compared to normotensive women [32].

Nitric oxide synthase 3 (NOS3) encoded for by *NOS3* (chromosome 7q36.1), is expressed predominantly in endothelial cells. NOS3 is responsible for maintaining vascular tone through synthesis or release of various endothelium-derived relaxing factors such as nitric oxide (NO) [45] and is thus important in BP regulation. Several reports state that reduced NO levels, resulting from polymorphisms in *NOS3*, may accompany preeclampsia and its severe forms [46]. For example, polymorphisms such as *NOS3* rs1799983G>T (c.894G>T; Glu298Asp), rs2070744T>C (c.−786T>C) and rs617220094a/4b (27-base pair VNTR in intron 4) have been shown to be among the usual culprits underlying susceptibility to preeclampsia in most African genomic studies. However, findings across African studies have been inconsistent (Supplementary Table S1).

Several studies report on associations of rs1799983G>T, rs2070744T>C and rs617220094a/4b with moderate to increased risk of preeclampsia among Tunisian, Egyptian and South African Mixed Ancestry women. Tunisian women carrying the rs2070744C allele were found to be at a significantly higher risk of preeclampsia compared to normotensive women [33]. Among Egyptian [34] and South African Mixed Ancestry women [35], rs1799983T allele was associated with an increased risk of preeclampsia. However, for Egyptian women, the effect was significant in the presence of the *UTS2* rs2890565G>A (c.221G>A; p.Ser74Thr) SNP.

The role of *GNB3*, encoding the G-protein β3 subunit of G-protein-coupled receptors (GPCRs), in regulating vascular function (i.e., vasoconstriction) [47] and in preeclampsia, was substantiated in a meta-analysis by Song et al., (2021) [48], particularly *GNB3* rs5443C>T (c.825C>T; p.Ser275=). In Africans, Tang et al., (2006) report on associations of rs5443T allele carriers with up to 2-fold odds (Figure 3A) of preeclampsia in African American women [36]. Furthermore, we would also like to note that the rs5443T allele occurs at frequencies up to 76% in Africans versus 51% in non-Africans [49], so even a modest effect, especially alongside other region-specific risk factors, may be clinically important.

### 4.2. Role of APOL1, ERAP2, HLA-G, IL-1β and TNF-α in Immunity and Susceptibility to Preeclampsia

Immune system dysregulation plays a major role in preeclampsia onset and progression. According to Mor et al., (2017), a balanced and favourable immune response is needed for successful implantation, while also protecting the foetus fatal immunological outcomes [50]. However, preeclampsia is characterised by an altered immune response that collectively leads to an activation of both innate and adaptive immune systems [51,52]. This leads to an increased circulation of immune system mediators such as macrophages, neutrophils, monocytes, natural killer (NK) cells, complement proteins, regulatory T cells, helper T cells and B cells [53,54] triggering widespread chronic inflammation and endothelial dysfunction [52].

During normal pregnancy, macrophages help establish the maternal–foetal interface by facilitating implantation and remodelling of the uterine spiral arteries [55,56]. Preeclampsia is characterised by an imbalance in the macrophage population (i.e., pro- and anti-inflammatory macrophages), leading to an increase in the production of pro-inflammatory cytokines such as IL-1α, IL-1β, TNF-α compared to anti-inflammatory cytokines such as IL-10 [57], whose underlying genetics have been shown to be associated with preeclampsia in Africans (Supplementary Table S1). However, effect sizes for SNPs in *TNF-α* (chromosome 6p21) and *IL-10* (chromosome 1q31–32) stand out the most (Figure 3B). Among Tunisian women, heterozygosity for the rs1799964C/T genotype and carriage of the rs1800750−rs1799964A−C haplotype was found to be associated with increased risk of preeclampsia. In further analyses, genotype and haplotype status seemed to correlate with TNF-α levels, suggesting that the SNPs mechanistically influence production of TNF-α [37]. Within the same population, carriers of the *IL-10* rs1800896–rs1800871–rs1800872A–T–A haplotype were associated with nearly double the odds of preeclampsia [38].

HLA-G facilitates differentiation of myeloid and T regulatory cells during normal pregnancy. This is essential for maternal–foetal immune tolerance [58]. Genetic variation in HLA-G in preeclampsia has not been widely explored, although there are several hypotheses stating that underlying genetic or epigenetic signals may be the significant contributing factors, leading to reduced HLA-G expression [59]. Among Africans, Loisel et al., (2013) reported on the contribution of *HLA-G* rs41557518ΔC to susceptibility to preeclampsia, and showed that African American women carrying the rs41557518ΔC allele were three times (Figure 3B) more likely to be at an increased of risk of preeclampsia [19].

*LEPR* located on chromosome 1p31.3 encodes the leptin receptor (LEPR) which is also expressed in the placenta [60]. LEPR is a target to the leptin hormone, which is responsible for the regulation of angiogenesis, smooth muscle proliferation and release of inflammatory mediators and cytokines [61,62]. Leptin levels have been shown to be high in preeclamptic women and rs1137101A>G (c.668A>G, p.Gln223Arg) and rs1805094G>T (c.1968G>T, p.Lys656Asn) have been reported to affect leptin levels [39]. In Sudanese women, rs1137101G allele and rs1137101-rs1805094G-G haplotype carriers were associated with significantly increased risk of preeclampsia (Figure 3B) [39].

*APOL1* (chromosome 22q12.3) encodes for the apolipoprotein L1, which has many biological functions (i.e., cholesterol or lipid transport) in addition to its role in the immune system. As a result, genetic variation in *APOL1* has been shown to be associated with increased risk of several diseases and has thus been investigated for its potential contribution to preeclampsia. To date, *APOL1* is one of the genes with the most studies in Africans that have reported preeclampsia risk, taking into account both maternal and foetal genetic variation (Supplementary Table S1, Table 1 and Figure 3B). With respect to maternal genetic variation, *APOL1* G1 [rs73885319A>G (c.1024A>G, p.Ser342Gly), rs60910145T>C (c.1098T>C, p.Ile366=)] was found to be associated with increased risk of EOPE in South African women [40]. Furthermore, a case report of an African–Colombian woman presenting with eclampsia, reported the presence of the *APOL1* G1/G2 (c.1164_1169del, p.Asn388_Tyr389del) high-risk genotype [31], linking these variants to severe forms of preeclampsia.

There are also reports providing evidence that carriage of the G1 and G2 variants by the foetus or offspring, increases the risk of developing preeclampsia in the mother [22,23,24]. Hong et al., (2020) report an African specific association of foetal *APOL1* G1 and G2 risk allele with increased susceptibility to preeclampsia, which appeared to be influenced by the country of origin of the mother [21]. This association seemed to be amplified nearly 3-fold especially if the mother and foetus had discordant genotypes [21], and this was confirmed by Miller et al., (2020) and Reidy et al., (2018) in separate cohorts of African American women [23,24] (Figure 3B). When we isolated and pooled variants in *APOL1* only, our analysis showed that the combined effect on overall preeclampsia susceptibility was 1.70-fold (pooled effect: OR (95% CI): 1.70 (1.39–2.07); *I*^2^: 0.0%, *p* = 0.51; GRADE: low certainty) and was suggestive of increased susceptibility (Figure 4).

*ERAP2* (chromosome 5q15) encodes the endoplasmic reticulum aminopeptidase 2 (ERAP2). ERAP2 not only regulates immune responses but is also involved in BP regulation and maintenance of normal pregnancy [41]. Altered placental ERAP2 expression has been reported to be characteristic of preeclampsia pregnancies in the first trimester, and Hill et al., (2011) demonstrates that foetal *ERAP2* rs2549782G>T (c.1041G>T, p.Lys347Asn) G allele significantly doubles the risk of preeclampsia in African American women [29].

### 4.3. Role of GLUT9, SLC4A1, SLCO4C1 and URAT1 in Cellular Homeostasis and Susceptibility to Preeclampsia

High uric acid levels have been reported to affect endothelial function and to inhibit foetal angiogenesis, leading to the manifestation of preeclampsia [63,64]. Polymorphisms in genes, particularly those leading to elevated levels of uric acid have been shown to augment preeclampsia risk [42]. To this effect, African genomic studies have highlighted roles of *GLUT9* (chromosome 4p16.1) and *URAT1* (chromosome 11q13.1) coding for the glucose transporter 9 and urate transporter 1, respectively, in preeclampsia (Figure 3C). In South African women, *URAT1* rs505802T>C (g.64589600T>C) C/T genotype seemed to be associated with higher risk of LOPE, while *GLUT9* rs1014290C>T (c.250-3296C>T) C/T genotype was more frequent in women with EOPE than normotensive women and associated with significantly higher risk of EOPE [42].

The placenta is of paramount importance in reproductive success as it mediates the exchange of biologically important compounds, particularly, nutrients, hormones or gases [65]. Therefore, transporters such as solute carriers (SLCs) are essential [66,67]. Morrison and colleagues [43] highlight the role of genetic variation in several SLCs and susceptibility to preeclampsia, also highlighting differences in the burden of polymorphisms between African and non-African women. African American women, carrying *SLC4A1* rs2074107G or rs2857078A and *SLCO4A1* rs10066650C alleles seemed to be at a significantly higher predisposition to preeclampsia, with nearly double the odds (Figure 3C), a finding attributed to altered activity of these transporters.

The genetic conflict hypothesis states that paternal genes (through the foetus), are selected to increase the transfer of nutrients across the placenta to the foetus, while maternal genes are selected to limit the transfer of nutrients to a level that balances the mother’s overall reproductive success [68]. Given the crucial role of transporters at the placenta, investigating how the foetal genetic variation in these transporters contributes to preeclampsia may be essential.

## 5. Discussion

We synthesised the available literature on genetic and epigenetic studies in women of African ancestry. Overall, we observed that the genomic factors underlying preeclampsia susceptibility in African populations have not been well characterised. In particular, we found that (i) studies on the contribution of maternal genetics to preeclampsia susceptibility remain limited and lack reproducibility across African populations (Supplementary Table S1), (ii) studies on the contribution of the foetal genome to preeclampsia susceptibility are largely based on African American populations rather than continental Africans (i.e., of African ancestry), and the spectrum of contributing genes investigated is currently limited (Table 1), and (iii) studies on the role of epigenetic factors in preeclampsia susceptibility are currently insufficient.

Although significant efforts have been made to determine the contribution of maternal genetic variation to preeclampsia susceptibility in African studies, there is a lack of reproducibility across African populations. Many studies seem to focus on different genes, possibly due to varying hypotheses or research questions, often leading to positive findings for a diverse array of genes (Supplementary Table S1). This is not surprising given the complex genetic architecture of preeclampsia compelling researchers to undertake this. But this means that evidence supporting a hypothesis in one population group is often not replicated in another, hindering the identification of strong likely causal candidate genes for African populations. For some genes, there are several reports available for more than one African population, but the findings or associations are different. *F5* rs6025G>A and *FII* rs1799963G>A, for instance, were studied in Tunisian and Sudanese populations and were shown to be associated with susceptibility to preeclampsia. However, the variants were found to be virtually non-existent in South Africans, suggesting that they play no role in preeclampsia risk in South Africa. This discrepancy likely reflects the extensive genetic diversity of African populations, but this lack of harmonisation still limits the identification of likely causal genes or variants.

Our synthesis also highlights that foetal genetics have not been fully explored in continental Africans. This is a critical gap, given that preeclampsia susceptibility has been attributed to genetic factors from the mother, foetus, and the couple at approximately 35%, 20%, and 13%, respectively [69]. To date, efforts in African women have mostly focused on African American populations and a single gene, *APOL1,* whose associations with preeclampsia as a risk factor show that it could be a strong candidate gene in preeclampsia [21,23,24,31,40]. However, these findings for *APOL1* have not been replicated in continental Africans, and this remains essential given that they increase maternal susceptibility to preeclampsia significantly. Other genes investigated in continental Africans (i.e., of African ancestry) examining the contribution of foetal genetics, such as *CD99*, *ERAP2*, *KIR*, *HLA-C*, *TNFR2*, *FII*, *F5*, *EPHX*, and *GSTP1*, have either shown no significant role or require further replication [18,25,26,27,29]. It is possible that they could be having significant effects in other African populations other than the ones they have been reported in so far.

Epigenetic modifications in preeclampsia have not been widely explored in African populations preeclampsia [70]. This area is still in its infancy, as only DNA methylation of the *MTHFR* gene has been investigated, as observed in a study among Nigerian women [71]. Although this is still an emerging area among Africans, it is possible that additional epigenetic signals associated with preeclampsia risk in many other genes remain to be discovered in African populations. We can already point out as an example, that there has been evidence of hypermethylation in some genes, such as *HLA-G*, which play a significant role in maternal immune tolerance during embryonic development [59]. However, whether *HLA-G* methylation status plays a role in preeclampsia, is a question still to be answered, particularly for African populations.

Compared to other candidate gene studies, genetic variation in *ERAP2* and *HLA-G*, for example, seemed to be consistent with studies performed in non-African populations [72,73]. While the populations are distinct, this may point to truly consistent signals across population groups in those genes.

**Figure 5:**
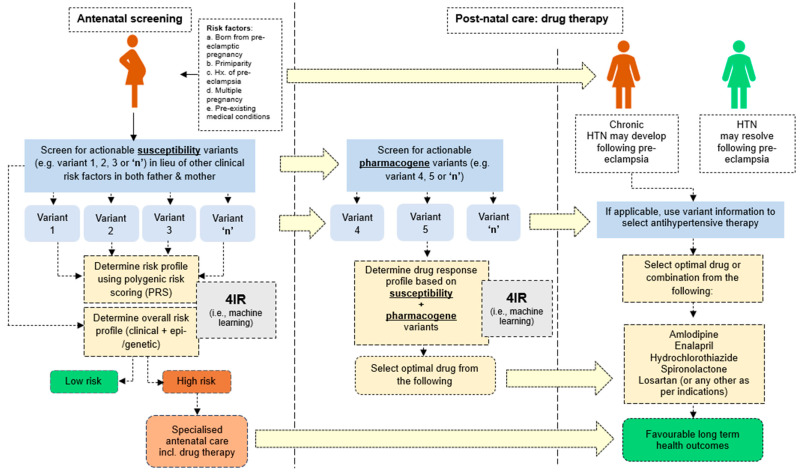
Proposed framework highlighting potential areas for incorporation of possible epi-/genetics in early detection and management of preeclampsia.

Traditionally, findings from genomic research are translated into genetic tests for clinical use; a process that is most practical for monogenic disorders. For more complex and polygenic disorders such as preeclampsia, there are usually many likely causal variants, which makes it less practical in genetic test development. In addition, the effect of these variants is usually confounded by many other non-genetic factors. Therefore, if genetic tests are to be developed for preeclampsia, there is also a need to account for several other confounding factors and using polygenic risk scores (PRSs) seem to be most practical in preeclampsia prediction or early detection. If such approaches could be further developed, especially for African populations, coupled to machine learning tools, this could facilitate early detection, management of preeclampsia and its subsequent long-term health outcomes (Figure 5).

However, the challenge that still persists, especially for African populations, is that there are no large-scale genome-wide association studies (GWASs) (from where PRS are generated) specific to preeclampsia [74]. This also adds on to the need for approaches beyond the common candidate gene approach. It is refreshing to notice that researchers have started to make progress towards this [75,76,77], but the need for such research in Africans remains critical. If comprehensive genomic data were harnessed for African populations, and variants with a strong evidence base were curated, the use of machine learning to develop multivariate predictive models incorporating these variants and other non-genetic factors represents a powerful approach that may significantly improve early detection, facilitating management of preeclampsia. Our meta-analysis seems to suggest that variants in *APOL1* (G1 or G2) and genes involved in vascular function, immune response/inflammation and cellular homeostasis may be a good place to start towards this effort. We thereby propose a theoretical diagnostic framework, highlighting the potential place of 4IR technologies such as machine learning, and role of genetic and/or epigenetic factors in preeclampsia detection, management and subsequent long-term outcomes (Figure 5).

### Limitations

We would like to acknowledge a key limitation in our core meta-analytic strategy of aggregating SNPs across different genes and loci into summary estimates. SNPs differ in allele frequency, linkage structure, functional impact and biological mechanism, so combining them into a single numeric “effect” may risk producing estimates that may not be biologically commensurate. Because of this, combined with GRADE ratings of low to very low, we present these pooled results as exploratory pathway-level summaries. Thus, our findings should be treated as a hypothesis generating, providing evidence for genes or pathways whose genes may need to be prioritised for further studies.

## 6. Concluding Remarks and Key Take Aways

The World Health Organisation’s (WHO) Sustainable Development Goals (SDG) aim to ensure healthy lives and promote well-being for all at all ages through a reduction in maternal mortality (Target 3.1) and non-communicable diseases (Target 3.4). Genomic factors may have utility in the early detection of preeclampsia and may potentially contribute to a reduction in both maternal and neonatal deaths. However, there are currently limited data on the genomic susceptibility factors underlying preeclampsia in populations of African descent. Existing studies are largely characterised by inconsistent findings, limited gene coverage, and an over-reliance on data from non-continental African groups, such as African Americans. Given the extensive genetic diversity among continental Africans, there is an urgent need for more comprehensive, harmonised research that specifically focuses on African populations.

## Figures and Tables

**Table 1:** Summary of genes reported in African studies considering the maternal and foetal genetic variation on susceptibility to preeclampsia and/or its subtypes.

Gene(s)	SNP(s)/Mutation(s)	Population	Mothers (N)	Babies (N)	Main Findings	Ref.
*CD99*	rs311103G>C	Ethiopian	241	241	rs311103C/C genotype associated increased PE risk in mothers carrying male foetuses	[18]
HLA-G *	rs1233334G>T, rs1630185G>A, rs41557518ΔC, rs12722482C>T, rs66554220I/D, rs1063320G>C	African American	372	372	Maternal rs41557518ΔC allele but not foetal was associated with increased PE risk and reduced serum levels of circulating HLA-G	[19]
F5 * FII *	rs6025G>A, rs1799963G>A	African American	374	369	No association observed between either maternal or foetal SNPs with PE or its severe forms	[20]
APOL1 *	G1 and G2	African American	328 1358 999 -	328 1358 999 672	Foetal G1/G2 associated with increased PE risk as well as differences in maternal and foetal genotypes, also implicated in preterm pregnancies and altered foetal growth	[21,22,23,24]
EPHX * *GSTP1*	rs1695A>G, rs1051740T>C	South African	345	300	Studied maternal and foetal polymorphisms not significantly associated with susceptibility to PE	[25]
KIR * HLA-C *	KIR haplotypes ^δ^ HLA-C epitopes ^ε^	Ugandan Ethiopian	738 288	738 288	Maternal KIR AA and foetal HLA-C alleles C2 epitope associated with increased PE risk in Ugandan and Ethiopian (for KIR AA only) women	[26,27]
TNFR2 *	rs1061622T>G	Tunisian	254	112	Maternal rs1061622G/G genotype associated with increased risk of preeclampsia	[28]
*ERAP2*	rs2549782T>G, rs17408150T>A	African American	799	837	Foetal rs2549782G allele but not rs17408150T>A was associated with increased risk for preeclampsia	[29]

SNP(s): single nucleotide polymorphism(s); N: total number of mother or baby in study; Ref: reference; PE: preeclampsia; CD99: cluster of differentiation 99 (cell migration, invasion and adhesion); ERAP2: endoplasmic reticulum aminopeptidase 2 (immune system regulation); GSTP1: glutathione S-transferase P1 (oxidative stress); HLA-C: human leukocyte antigen C (immune system regulation); KIR: killer-cell immunoglobulin-like receptor (immune system regulation); TNFR2: tumour necrosis factor receptor 2 (immune system regulation); * genes also reported in studies on maternal genetics only, refer to Supplementary Table S1 for their roles; ^δ^ denotes centromeric (cA and cB) and telomeric (tA and tB) haplotypes and ^ε^ denotes C1 and C2 epitopes.

**Table 2:** Summary of functional significance of polymorphisms that are most significant for preeclampsia in published African studies.

Gene	SNP ID	dbSNP Annotation	Effect Allele	Functional or Predicted Impact	Direction of Effect
*FLT1*	rs12584067G>C (c.3287-523G>C) rs7335588C>G (c.1437-4471C>G)	intronic	G G	may affect gene expression or splicing, leading to altered FLT1 levels and vascular dysfunction	↑
*VEGFA*	rs3025039C>T (c.*237C>T)	3′-UTR	T	may affect gene expression, leading to altered placental development and endothelial dysfunction	↑
*VEGFC*	rs1485766A>C (c.705-1803A>C) rs6838834C>T (c.148-2698G>A)	intronic	A C	may affect gene expression or splicing, leading to angiogenic imbalance and endothelial dysfunction	↑
*NOS3*	rs1799983G>T (c.894G>T; Glu298Asp) rs2070744T>C (c.−786T>C)	missense 5′-UTR	T C	alters protein conformation and leads to reduced nitric oxide bioavailability, a vasodilator alters transcription efficiency and leads to reduced nitric oxide bioavailability	↑
*GNB3*	rs5443C>T (c.825C>T; p.Ser275=)	synonymous	T	causes alternative splicing, leading to increased G-protein and vascular reactivity	↑
*HLA-G*	rs41557518ΔC	frameshift	delC	alters protein expression, leading to formation of a non-functional protein	↑
*IL1α*	rs17561G>T (c.340G>T; p.Ala114Ser)	missense	G	may affect levels of inflammatory markers, potentially leading to systemic inflammation	↑
*IL1β*	rs16944T>C (g.112837290T>C) rs1143634 C>T (c.315C>T, p.Phe105=)	intron synonymous	C T	may affect levels of inflammatory markers, potentially leading to systemic inflammation	↑
*LEPR*	rs1805094G>C (c.1968G>T, p.Lys656Asn) rs1137101A>G (c.668A>G, p.Gln223Arg)	missense missense	G G	alter protein conformation and leptin receptor signalling function	↑
*APOL1*	rs60910145T>C (c.1098T>C, p.Ile366=) rs73885319A>G (c.1024A>G, p.Ser342Gly)	missense missense	G C	alters protein conformation and induces damage to endothelial cells as a result of membrane pore formation triggering increased inflammation, mitochondrial dysfunction and impaired autophagy	↑
*ERAP2*	rs2549782G>T (c.1041G>T, p.Lys347Asn)	missense	G	alters protein conformation and substrate specificity affecting antigen processing by the immune system	↑
*AGT*	rs699C>T (c.776T>C, p.Met259Thr) rs4762C>T (c.593C>T, p.Thr198Met)	missense missense	C C	affects stability and expression of angiotensinogen, leading to altered blood pressure regulation (C allele for rs4762 protective in preeclampsia)	↑ ↓
*SLC4A1*	rs2074107G>A (g.44260608G>A) rs2857078A>C (g.44252803A>C)	intronic intronic	G A	may affect gene expression or splicing, leading to altered membrane transport and endothelial dysfunction	↑
*SLCO4A1*	rs10066650A>C (c.1470-1882A>C)	intronic	C	may affect gene expression or splicing, leading to altered uptake of organic anions needed for maintaining renal homeostasis	↑
*TNF-α*	rs1799964T>C (c.−1031T>C)	5′-UTR	C	may affect gene expression, potentially leading to chronic, low-grade inflammation	↑

↑ denotes high risk and ↓ denotes low risk (or protective).

## Data Availability

No new data were created or analyzed in this study. Data sharing is not applicable to this article.

## Supplementary Materials

The following supporting information can be downloaded at: https://www.mdpi.com/article/10.3390/ijms27062594/s1. Refs. [78,79,80,81,82,83,84,85,86,87,88,89,90,91,92,93,94,95,96,97,98,99,100,101,102,103,104,105,106,107,108,109,110,111,112,113,114,115,116] are cited in the Table S1.

## Abbreviations

The following abbreviations are used in this manuscript:

4IR	Fourth Industrial Revolution
ML	Machine Learning
BP	Blood Pressure
DBP	Diastolic Blood Pressure
SBP	Systolic Blood Pressure
GRADE	Grading of Recommendations, Assessment, Development and Evaluation
ISHHP	International Society for the Study of Hypertension in Pregnancy
AKI	Acute Kidney Injury
SNP	Single Nucleotide Polymorphisms
GWAS	Genome-wide Association Study
PRS	Polygenic Risk Score
WHO	World Health Organisation
SDG	Sustainable Development Goals
