# Supplementary material for: Preeclampsia Genomic Susceptibility Factors in Populations of African Ancestry: A Systematic Review and Meta-Analysis
Source: Int J Mol Sci. 2026 Mar 12;27(6):2594. doi: 10.3390/ijms27062594 (PMC13027360; doi:10.3390/ijms27062594)
Supplement: Supplementary file 1 [file ijms-27-02594-s001.zip › Summary of search terms.pdf]

Summary of search terms

“Pre-eclampsia AND genetics AND Africa” OR “Genetics of pre-eclampsia in Africans” OR “Single nucleotide polymorphisms AND pre-eclampsia AND Africans” OR “Genetic variation AND pre-eclampsia AND Africans” OR “Genetics AND hypertension in pregnancy AND Africa” OR “Pre-eclampsia AND candidate gene AND GWAS AND Africa” OR “Pre-eclampsia AND epigenetics AND Africans” OR “Pre-eclampsia AND foetal genetics AND paternal genetics AND Africans”.
