# Supplementary material for: Preeclampsia Genomic Susceptibility Factors in Populations of African Ancestry: A Systematic Review and Meta-Analysis
Source: Int J Mol Sci. 2026 Mar 12;27(6):2594. doi: 10.3390/ijms27062594 (PMC13027360; doi:10.3390/ijms27062594)
Supplement: Supplementary file 1 [file ijms-27-02594-s001.zip › Supplementary Figure S1.pdf]

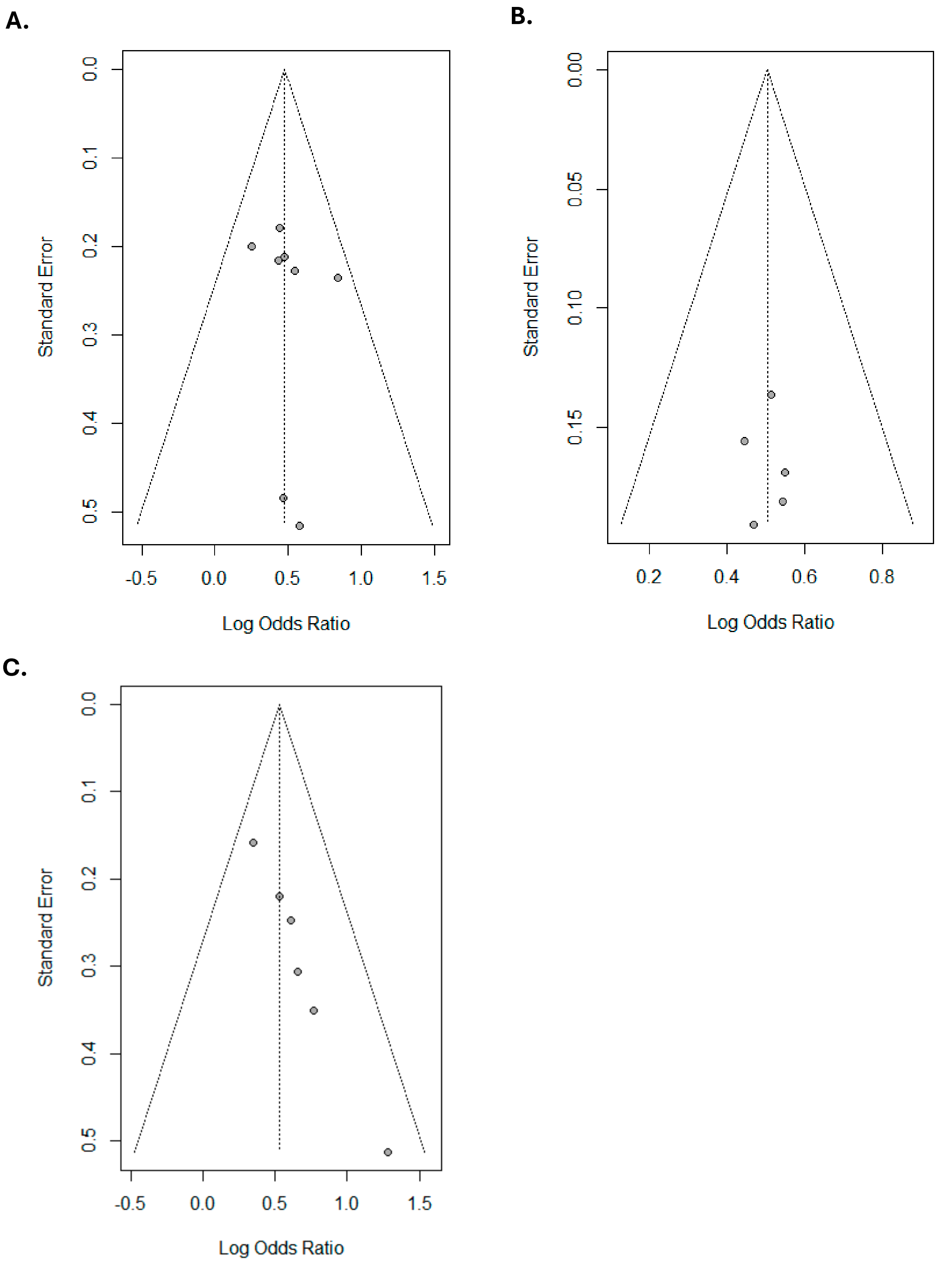

Supplementary Figure S1: Funnel plots assessing publication bias when SNPs affecting (**A**) vascular function (**B**) cellular homeostasis and (**C**) *APOL1* were pooled together.
