# Supplementary material for: Preeclampsia Genomic Susceptibility Factors in Populations of African Ancestry: A Systematic Review and Meta-Analysis
Source: Int J Mol Sci. 2026 Mar 12;27(6):2594. doi: 10.3390/ijms27062594 (PMC13027360; doi:10.3390/ijms27062594)
Supplement: Supplementary file 1 [file ijms-27-02594-s001.zip › Supplementary Figure S2.pdf]

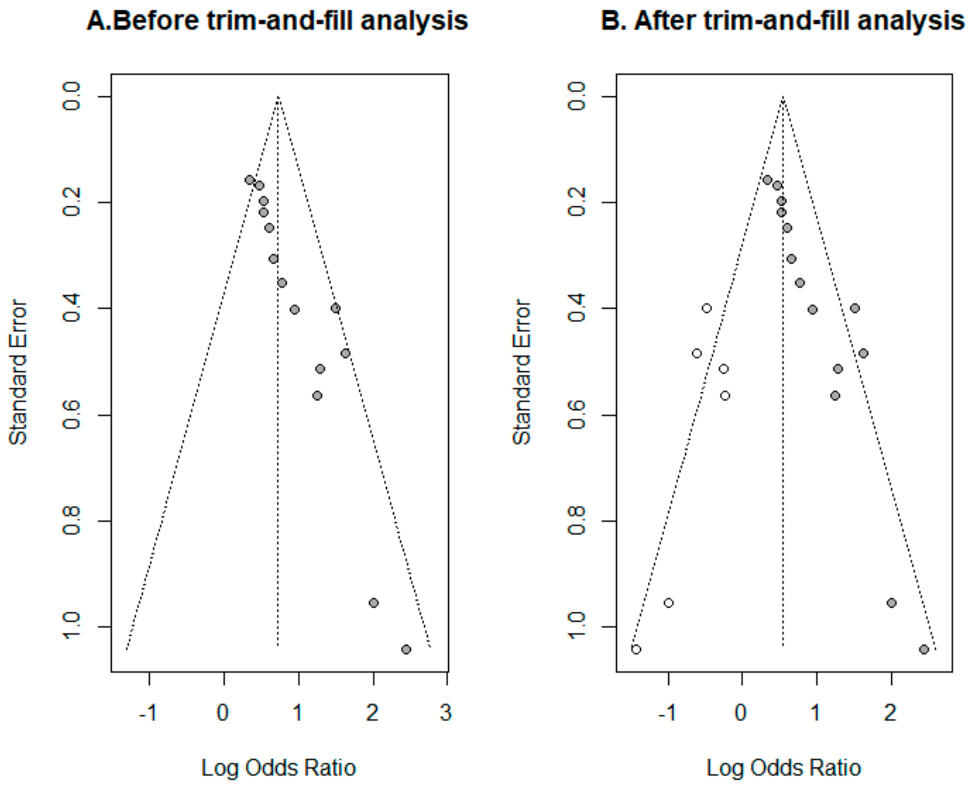

Supplementary Figure S2: (A) Publication bias assessment for SNPs affecting immune response/inflammation before trim-and-fill analysis and (B) after trim-and-fill analysis – 6 studies were imputed (unshaded circles).
