# Supplementary material for: Preeclampsia Genomic Susceptibility Factors in Populations of African Ancestry: A Systematic Review and Meta-Analysis
Source: Int J Mol Sci. 2026 Mar 12;27(6):2594. doi: 10.3390/ijms27062594 (PMC13027360; doi:10.3390/ijms27062594)
Supplement: Supplementary file 1 [file ijms-27-02594-s001.zip › Supplementary Table S1.pdf]

Supplementary Table S1: Summary of genes reported in African studies and their influence on susceptibility to pre-eclampsia and/or its subtypes

Gene(s)	Enzyme/protein	Pathway/ System	SNP(s)/mutation(s)	Population (and sample size)	Main Findings	Ref.
VEGFA	Vascular endothelial growth factor A	Endothelial	rs833052C>A, rs699947C>A, rs833061C>T, rs1570360A>G, rs2010963G>C, rs25648C>T, rs833068G>A, rs833070C>T, rs3025020C>T, rs3025039C>T	Tunisian (600)	No significant associations observed individual SNPs; haplotypes ATGCCAA, ACAGCAG and CCAGCGG associated with increased PE risk while CCAGCAA and ATGCCGG were protective	[78] [a]
VEGFA	Vascular endothelial growth factor A	Endothelial	rs3025039C>T, rs699947C>A	Sudanese (120), Ugandan (250), Egyptian (290)	rs3025039T associated with increased PE risk in Sudanese but not Ugandan women; no association observed for rs699947C>A in Egyptian women	[79,80]
VEGFC	Vascular endothelial growth factor C	Endothelial	rs1485766A>C, rs6838834C>T	African American (606)	rs1485766A and rs6838834C were associated with increased risk of PE	[32]
NOS3	Nitric oxide synthase	Endothelial	rs1799983G>T, rs2070744T>C, rs617220094a/4b	Tunisian (634), Ghanaian (150 & 220), Egyptian (53), South African (150)	rs2070744C allele associated with increased PE risk in Tunisians; rs61722009 4b/4b associated with reduced PE risk; rs1799983T allele associated with increased PE risk in Egyptian and South African MA women	[36-38,81,82]
FGF1<br>*FGF2*	Fibroblast growth factor 1<br>Fibroblast growth factor 2	Angiogenesis	rs34011C>T<br>rs2922979C>G	Tunisian (600)	rs34011T allele was associated with increased PE risk	[83]
MTHFR	Methylenetetrahydrofolate reductase	Folate metabolism	rs1801133C>T, rs1801131A>C	Sudanese (320), Nigerian (400), Zimbabwean (356), South African (215 and 687)	rs1801133T allele associated with increased PE risk in Sudanese and Nigerian women but not Zimbabwean and South African women; 1317T/T was protective of PE in South African women	[84-88]
MTR	Methionine synthase	Folate metabolism	rs1805087A>G	Nigerian (400)	rs1805087G allele associated with increased PE risk	[88]
F5<br>*FII*	Factor V Leiden<br>Prothrombin	Thrombosis/ Coagulation	rs6025G>A<br>rs1799963G>A	Sudanese (100), South African (220)	rs6025A allele associated with increased PE risk in Sudanese women, rs6025G>A and rs1799963G>A were not detected in Zulu speaking South Africans	[89-91]
TM	Thrombomodulin	Thrombosis/ Coagulation	rs1042579G>A	South African (220)	rs1042579A allele detected in 1.5% Zulu speaking South Africans but no association with PE observed	[91]
LEPR	Leptin receptor	Immune response	rs1137101A>G, rs1805094G>C	Sudanese (244)	rs1137101G allele associated with increased PE risk; rs1137101-rs1805094G-G haplotype associated with increased PE risk	[58]
FAS<br>*FASL*	TNF-receptor superfamily	Immune response	rs1800682A>G<br>rs5030772A>G	Egyptian (100)	rs1800682G was associated with increased PE risk, rs5030772G allele associated with reduced PE risk	[92]
ENG<br>*TGFβR1*<br>*TGFβR2*	Endoglin receptor<br>Transforming growth factor beta receptor 1 and 2	Angiogenesis	rs11792480G>A, rs10739778A>C,rs6550005G, rs1346907C>T, rs877572G>C	Egyptian (125), African American (415)	rs10739778C/C associated with reduced PE risk in Egyptian women; rs10739778A, rs6550005A, rs1346907C and rs877572G alleles associated with increased PE risk in African Americans	[93,94]
VDR	Vitamin D receptor	Vitamin D metabolism	rs2228570(Fok1), rs1544410(Bsm1)	Ghanaian (162)	"bb" genotype associated with reduced PE risk	[81]
UTS2	Urotensin II	Inflammation	rs2890565G>A	Egyptian (118)	rs2890565A allele associated with increased PE risk	[37]
IL1β	Interleukin 1 beta	Immune response	rs16944T>C, rs1143634 C>T	Sudanese (120)	rs16944C/T associated with increased PE risk	[79]

SCNN1B	Beta-subunit of the epithelial sodium channel	Sodium Homeostasis	rs149868979G>A, T594M	South African (428 and 729)	rs149868979G>A mutation associated with increased PE risk; no association observed for T594 mutation.	[95,96]
TGF- β1	Transforming growth factor β1	Immune response	codon 10<br>codon 25	Zimbabwean (172)	T allele in codon 10 more frequent in women with PE, may be associated with PE and eclampsia	[97]
GNB3	Guanine nucleotide-binding protein subunit β3	Signalling pathway	rs5443C>T	African American (349)	rs5443T allele frequent in PE women and associated with increased PE risk	[41]
SLC4A1<br>*SLCO4C1*	Solute carrier family 4 member 1<br>Solute carrier organic anion transporter family member 4C1	Homeostasis	rs2857078A>C, rs2074107G>A, rs10066650C>A	African American (631)	rs2857078A, rs2074107G and rs10066650C allele associated with increased PE risk	[67]
EMMPRIN	Extracellular matrix metalloproteinases inducer	Placentation	rs424243T>G	Ghanaian (140)	rs424243G/T associated with increased PE risk and decreased MMP-2 activity	[98]
FLT1	fms-like tyrosine kinase 1	Endothelial	rs12584067C>G, rs7335588C>G	African American (606)	rs12584067G and rs7335588G allele associated with increased PE risk	[32]
APOL1	Apolipoprotein L1	Lipid metabolism	G1 (rs7388319A>G, rs60910145T>C)<br>G2 (rs71785313I/D)	Afro-Colombian (1), South African (428)	G1 and G2 implicated in eclamptic seizures in Afro-Colombian woman; G1 associated with increased EOPE risk in South African women	[31,59]
APOE	Apolipoprotein E	Lipid metabolism	rs429358T>C, rs7412C>T	South African (213)	No significant association but varepsilon 2/2 genotype associated with an increase in perinatal deaths	[99]
PAR4	Platelet protease activated receptor 4	Thrombosis/ Coagulation	rs773902G>A	African American (320)	No significant association observed but rs773902A allele appeared to increase risk of preterm birth	[30]
HLA-G	Human leukocyte antigen-G	Immune response	rs1707T>C, rs66554220Ins/Del, rs1710G>C, rs1063320G>C, rs9380142A>G, rs1610696G>C	South African (193)	rs66554220Ins/Del, rs9380142A>G, rs1610696G>C, and rs1707T>C associated with increased PE risk in HIV infected women	[100]
C1q	Complement component 1q	Immune response	rs292001G>A, rs294183C>T	South African (325)	rs292001A/G associated with susceptibility to PE, irrespective of HIV status	[101]
LGALS13	Placental protein 13	Trophoblast invasion	Exon 1 – 4	African American (263)	rs3764843A/A associated with reduced LGALS13 expression and PE risk in the first trimester	[102]
ANPEP<br>*LNPEP*	Aminopeptidase N<br>Leucyl-N-exopeptidase	RAAS	rs6496603A>G, rs18059C>A	South African (637)	No significant association observed	[103]
PRCP<br>*ACE*<br>*AGT*	Prolyl carboxypeptidase<br>Angiotensin-converting enzyme<br>Angiotensin	RAAS	rs2298668T>G<br>rs4459610A>T, rs1799752I/D<br>rs699C>T, rs4762G>A, rs5049G>A	Ghanaian (220), African American (1165 & 783), South African (603, 623 & 150)	PRCP rs2298668G associated with increased PE risk and reduced PRCP transcript levels in African Americans; no association for rs4459610A>T, rs1799752I/D and rs5049G>A in South Africans and African Americans; rs699T allele associated with increased PE risk in South African Zulu women and no association for Ghanaian, African American and South African MA women	[38,82,104-107]
ACTN4<br>*NPHS1*	Alpha-actinin-4<br>Nephrin	Homeostasis/ sodium	rs121908415A>G, rs3745859C>T, rs74315346A>G, rs869025495C>T, rs437168C>T	South African (637)	rs437168C allele carriers associated with increased PE and EOPE risk	[108]
URAT1<br>*PDZK1*<br>*GLUT9*	Urate transporter 1<br>PDZ domain containing 1<br>Glucose transporter 9	Uric acid metabolism	rs505802C>T, rs12129861C>T, rs1014290A>G	South African (637)	rs505802C/T and rs1014290C/T associated with increased PE risk	[63]
MT-ND5	Mitochondrial NADH dehydrogenase 5	Oxidative phosphorylation	rs2853502A>T	African American (68)	Nonsynonymous substitutions in mtDNA particularly rs2853502A>T may be risk factors for PE	[109]
AT1R<br>*AT2R*	Angiotensin II type-1 receptor<br>Angiotensin II type-2 receptor	RAAS	rs5186A>C, rs14035430A>G, rs11091046A>C	Afro-Caribbean (662), South African (603)	rs14035430G/G more frequent in women with PE than normotensives for Afro-Caribbeans; no association observed in South African women	[105,110]

miR-146a	MicroRNA-146a	Immune response	rs2910164G>C	South African (193)	rs2910164C allele protective of severe PE	[111]
IL-10	Interleukin-10	Immune response	rs1800872C>A, rs1800871C>T, rs1800896A>G	Tunisian (645), African American (811)	rs1800871T allele and rs1800872–rs1800871–rs1800896A –T –A haplotype associated with increased PE risk in Tunisian women	[52,112]
IL-1α<br>*IL-1β*	Interleukin-1 alpha<br>Interleukin-1 beta	Immune response	rs17561G>T, rs1800587C>T, –3957C>T	African American (811)	rs17561G/G and rs1800587C/C associated with increased PE risk	[112]
TNF-α	Tumour necrosis factor-α	Immune response	rs1800629G>A, rs1799964T>C, rs1800750G>A, rs361525G>A, rs80267959G>A	African American (811), Tunisians (600)	No association for rs1800629G>A in African Americans; rs1799964C allele and rs1799964–rs1800750C–A haplotype associated with increased PE risk Tunisian women	[51,112]
REN	Renin	RAAS	rs12750834C>A	South African (603)	No significant association observed	[105]
ICAM-1<br>*VCAM-1*<br>*SEL-E*	Intercellular adhesion molecule-1<br>Vascular adhesion molecule-1<br>E-selectin	Immune response	rs3093030C>T, rs3783605A>G, rs1805193A>C	South African (405)	rs3093030T and rs3783605G alleles but not rs1805193A>C associated with increased PE risk	[113]
EPHX1	Epoxide hydrolase 1	Oxidative stress	rs1051740T>C, rs2234693T>C	Ghanaian (220)	rs1051740T allele associated with increased severe PE risk	[82]
CYP11B2	Aldosterone synthase	RAAS	rs1799998C>T	South African (623)	rs1799998T/T protective of PE	[106]
CORIN	Atrial natriuretic peptide-converting enzyme	Homeostasis/ sodium	rs2271036, rs2271037	Morocco, Algeria, Tunisia (571[b])	rs2271036C and rs2271037G alleles associated with increased PE risk	[114]
ENPEP	Aminopeptidase A	RAAS	rs6825911C>T	South African (602)	No significant association	[115]
GC	Vitamin D binding protein	Vitamin D metabolism	rs4588C>A, rs7041T>G	South African (600)	rs4588C/C and rs7041T/T associated with increased PE risk	[116]

SNPs: single nucleotide polymorphisms; Ref: reference; PE: pre-eclampsia; EOPE: early onset pre-eclampsia, RAAS: renin-angiotensin aldosterone system, HIV: human immunodeficiency virus; MMP-2: matrix metalloproteinase-2; MA: Mixed Ancestry; [a] the haplotype SNP combinations is in this order: rs833052C>A, rs699947C>A, rs833061C>T, rs1570360A>G, rs2010963G>C, rs25648C>T, rs833068G>A, rs833070C>T, rs3025020C>T, rs3025039C>T; [b] individuals from Sub-Saharan Africa also included, but not specified.
