# Supplementary material for: Preeclampsia Genomic Susceptibility Factors in Populations of African Ancestry: A Systematic Review and Meta-Analysis
Source: Int J Mol Sci. 2026 Mar 12;27(6):2594. doi: 10.3390/ijms27062594 (PMC13027360; doi:10.3390/ijms27062594)
Supplement: Supplementary file 1 [file ijms-27-02594-s001.zip › Supplementary Table S2.pdf]

Supplementary Table S2: Study quality assessment using the CASP checklist for case-control studies

Author, Year	Did the study address a clearly focused issue?	Did the authors use an appropriate method to answer their question?	Were the cases recruited in an acceptable way?	Were the controls selected in an acceptable way?	Was the exposure accurately measured to minimize bias?	Aside from the exposure, did the groups have similar characteristics?	Have the authors taken account of the potential confounding factors in the design and/or in their analysis?	Was the treatment effect large?	Was the estimate of the treatment effect precise?	Do you believe the results?	Can the results be applied to your patients/the population of interest?	Do the results of this study fit with other available evidence?	% Score
Gannoun et al 2015	5	5	3	5	0	5	5	5	5	5	5	5	88
Gannoun et al 2018	5	5	5	5	4	5	5	5	0	4	0	5	80
Marwa et al 2016	5	5	5	5	0	5	5	0	5	5	5	0	75
Fondjo et al 2023	5	5	2	2	0	5	0	5	5	0	0	5	57
Ahmed et al 2020	5	5	5	5	5	5	4	4	0	5	5	5	88
Elzein et al 2020	5	5	3	3	0	5	0	3	0	0	0	5	48
Saad et al 2020	5	5	5	5	5	5	5	5	5	5	5	5	100
Nasr et al 2014	5	5	3	3	0	5	0	3	3	3	5	5	67
Mowad et al 2019	5	5	5	5	0	5	0	0	0	0	0	0	42
ElMonier et al 2019	5	5	3	3	0	5	0	3	3	3	5	5	67
Fondjo et al 2024	5	5	4	4	0	5	0	3	3	2	2	0	55
Kelemu et al 2020	5	5	4	5	3	0	0	0	0	0	0	0	37
El-Sherbiny et al 2013	5	5	3	4	0	5	5	5	5	5	3	5	83
Hamid et al 2020	5	5	5	5	5	5	5	5	5	5	5	5	100
Khaliq et al 2020	5	5	5	5	5	5	0	2	3	3	5	3	77
Aung et al 2017	5	5	5	5	3	0	0	2	3	3	5	5	68
Thakoordeen-Reddy et al 2020	5	5	4	4	3	5	0	3	3	3	5	5	75
Sibiya et al 2024	5	5	5	5	3	5	0	2	2	2	2	2	63
Groten et al 2014	5	5	4	5	4	5	5	5	5	5	5	5	97
Pegoraro et al 2004	5	5	4	5	5	0	0	2	2	2	3	5	63
Aung et al 2018	5	5	5	5	3	5	0	2	2	2	2	5	68
Bell et al 2013	5	5	5	5	5	5	5	5	3	4	3	3	88
Hill et al 2011	5	5	5	5	3	5	5	5	5	5	3	5	93
Jenkins et al 2008	5	5	3	5	5	5	2	0	0	2	2	5	65
Aung et al 2021	5	5	4	5	3	5	0	0	0	0	0	0	45
Haggerty et al 2005	5	5	4	5	5	5	5	5	3	4	3	4	88
Miller et al 2020	5	5	5	5	4	0	5	3	5	5	5	5	87
Osunkalu et al 2020	5	5	4	5	0	0	0	2	2	2	2	5	53

Raguema et al 2022	5	5	5	5	0	5	5	3	5	5	3	4	83
Naidoo et al 2019	5	5	5	5	3	5	0	2	0	0	2	3	58
Nwabeyambo et al 2023	5	5	5	5	5	5	5	4	5	5	4	5	97
Hillerman et al 2005	5	5	3	3	2	5	0	3	3	3	4	5	68
Said et al 2013	5	5	4	4	2	0	0	3	3	3	3	3	58
Zitouni et al 2018	5	5	5	5	2	5	5	5	5	5	3	5	92
Osunkalu et al 2021	5	5	3	4	2	0	2	0	0	3	3	5	53
Gerhardt et al 2004	5	5	4	5	2	0	0	2	2	3	5	3	60
Reidy et al 2018	5	5	5	5	4	0	5	3	5	4	3	5	82
Phoswa et al 2020	5	5	3	3	2	0	0	3	0	3	3	3	50
Nakimuli et al 2015	4	5	3	4	4	0	0	3	0	2	3	4	53
Govender et al 2023	4	5	4	4	4	0	2	4	5	2	3	4	68
Madar-Shapiro et al 2018	4	5	4	4	4	0	4	4	5	4	3	4	75
Khaliq et al 2020	4	5	4	4	3	0	2	3	5	3	3	4	67
Wang et al 2006	4	5	4	4	4	0	4	5	3	4	4	4	75
Khaliq et al 2021	4	4	4	4	4	0	2	2	5	3	4	4	67
Pegoraro et al 2004	4	4	4	4	4	0	2	3	5	3	4	4	68
Ding et al 2012	4	4	4	4	2	0	2	5	3	3	3	4	63
Kelemu et al 2020	4	4	4	4	3	0	2	3	5	3	3	4	65
Akbar et al 2009	4	4	4	4	4	0	4	5	5	4	4	4	77
Maharaj et al 2017	4	4	4	4	4	0	2	0	0	3	4	4	55
Raguema et al 2018	4	4	4	4	4	0	4	3	5	4	4	4	73
Ahmed et al 2019	5	5	5	5	5	3	4	4	3	5	5	5	90
Loisel et al 2013	5	5	5	5	5	5	0	5	5	5	5	5	92
Stanczuk et al 2007	5	5	5	4	0	5	0	0	0	0	0	3	45
Tang et al 2006	5	5	5	5	0	5	0	3	5	5	5	5	80
Morrison et al 2010	5	5	5	0	5	3	5	3	5	5	5	3	82
Amakye et al 2023	5	5	5	5	5	5	0	5	3	5	5	3	85
Srinivas et al 2010	5	5	5	5	5	5	5	3	5	5	5	3	93
Livingston et al 2001	5	5	5	5	0	4	0	0	0	5	0	2	52
Rajkovic et al 2000	5	5	5	5	0	5	5	0	5	5	5	5	83
Chikosi et al 2000	5	5	3	3	0	4	0	0	3	5	0	0	47
Hong et al 2021	5	5	5	5	3	5	5	4	5	5	5	3	92
Azhibekov et al 2023	5	5	3	3	0	0	5	4	5	5	5	5	75
