# Supplementary material for: Preeclampsia Genomic Susceptibility Factors in Populations of African Ancestry: A Systematic Review and Meta-Analysis
Source: Int J Mol Sci. 2026 Mar 12;27(6):2594. doi: 10.3390/ijms27062594 (PMC13027360; doi:10.3390/ijms27062594)
Supplement: Supplementary file 1 [file ijms-27-02594-s001.zip › Supplementary Table S3.pdf]

Supplementary Table S3: Study quality assessment using the CASP checklist for cohort studies

Author, Year	Did the study address a clearly focused issue?	Was the cohort recruited in an acceptable way?	Was the exposure accurately measured to minimise bias?	Was the outcome accurately measured to minimise bias?	Have the authors identified all important confounding factors?	Have they taken account of the confounding factors in the design and/or analysis?	Was the follow up of subjects complete enough?	Was the follow up of subjects long enough?	What are the results of this study?	How precise are the results?	Do you believe the results?	Can the results be applied to the local population?	Do the results of this study fit with other available evidence?	What are the implications of this study for practice?	% Score
Boelig et al 2022	5	4	3	5	5	5	5	5	3	3	5	3	4	3	83
