# Supplementary material for: Preeclampsia Genomic Susceptibility Factors in Populations of African Ancestry: A Systematic Review and Meta-Analysis
Source: Int J Mol Sci. 2026 Mar 12;27(6):2594. doi: 10.3390/ijms27062594 (PMC13027360; doi:10.3390/ijms27062594)
Supplement: Supplementary file 1 [file ijms-27-02594-s001.zip › Supplementary Table S4.pdf]

Supplementary Table S4: Study quality assessment using the JBI checklist for case reports

Author, Year	Were patient's demographic characteristics clearly described?	Was the patient's history clearly described and presented as a timeline?	Was the current clinical condition of the patient on presentation clearly described?	Were diagnostic tests or assessment methods and the results clearly described?	Was the intervention(s) or treatment procedure(s) clearly described?	Was the post-intervention clinical condition clearly described?	Were adverse events (harms) or unanticipated events identified and described?	Does the case report provide takeaway lessons?	% Score
Duran et al 2024	5	5	5	4	5	5	5	5	98
