# Supplementary material for: Preeclampsia Genomic Susceptibility Factors in Populations of African Ancestry: A Systematic Review and Meta-Analysis
Source: Int J Mol Sci. 2026 Mar 12;27(6):2594. doi: 10.3390/ijms27062594 (PMC13027360; doi:10.3390/ijms27062594)
Supplement: Supplementary file 1 [file ijms-27-02594-s001.zip › Supplementary Table S5.pdf]

Supplementary Table S5: Summary of the GRADE evaluation

	Risk of Bias	Inconsistency	Imprecision	Publication bias	OR (95% CI)	Certainty of evidence (GRADE)
**Subgroup A Vascular function**	No	No	No	Yes	1.61 (1.38 – 1.88)	Low
**Subgroup B Immune response/inflammation**	No	No	No	Yes	2.07 (1.68 – 2.54)	Low
**Subgroup C Cellular homeostasis**	No	No	No	No	1.65 (1.41 – 1.91)	Low
***APOL1* G1/G2 only**	No	No	No	Yes	1.70 (1.39 – 2.07)	Low
