# Supplementary material for: Preeclampsia Genomic Susceptibility Factors in Populations of African Ancestry: A Systematic Review and Meta-Analysis
Source: Int J Mol Sci. 2026 Mar 12;27(6):2594. doi: 10.3390/ijms27062594 (PMC13027360; doi:10.3390/ijms27062594)
Supplement: Supplementary file 1 [file ijms-27-02594-s001.zip › Supplementary Table S6.pdf]

Supplementary Table S6: Leave one out analysis vascular function

Omitted study	OR	95% CI	p-value	τ^2	τ	I^2
Omitting Gannoun et al 2015a	1.6755	[1.4208; 1.9759]	< 0.0001	0	0	0%
Omitting Gannoun et al 2015b	1.6118	[1.3817; 1.8803]	< 0.0001	0	0	0%
Omitting Groten et al 2014	1.5965	[1.3582; 1.8767]	< 0.0001	0	0	0%
Omitting Nwabeyambo et al 2023	1.6076	[1.3784; 1.8748]	< 0.0001	0	0	0%
Omitting Srinivas et al 2010a	1.6233	[1.3714; 1.9216]	< 0.0001	0	0	0%
Omitting Srinivas et al 2010b	1.6205	[1.3769; 1.9073]	< 0.0001	0	0	0%
Omitting Srinivas et al 2010c	1.6205	[1.3769; 1.9071]	< 0.0001	0	0	0%
Omitting Srinivas et al 2010d	1.6115	[1.3685; 1.8976]	< 0.0001	0	0	0%
Omitting Tang et al 2006	1.5418	[1.3125; 1.8110]	< 0.0001	0	0	0%
**Random effects model**	**1.6113**	**[1.3840; 1.8759]**	**< 0.0001**	**0**	**0**	**0%**
