# Supplementary material for: Preeclampsia Genomic Susceptibility Factors in Populations of African Ancestry: A Systematic Review and Meta-Analysis
Source: Int J Mol Sci. 2026 Mar 12;27(6):2594. doi: 10.3390/ijms27062594 (PMC13027360; doi:10.3390/ijms27062594)
Supplement: Supplementary file 1 [file ijms-27-02594-s001.zip › Supplementary Table S7.pdf]

Supplementary Table S7: Leave one out analysis immune system and inflammation SNPs

Omitted study	OR	95% CI	p-value	τ^2	τ	I^2
Omitting Saad et al 2020	2.0131	[1.6431; 2.4663]	< 0.0001	0.0434	0.2083	43.5%
Omitting Haggerty et al 2005	1.9992	[1.6429; 2.4328]	< 0.0001	0.0387	0.1967	38.1%
Omitting Haggerty et al 2005b	1.8915	[1.5909; 2.2488]	< 0.0001	0.0168	0.1298	33.5%
Omitting Hamid et al 2020b	2.0533	[1.6532; 2.5502]	< 0.0001	0.0549	0.2342	45.0%
Omitting Hamid et al 2020c	1.8288	[1.5627; 2.1402]	< 0.0001	0.0055	0.0744	30.9%
Omitting Thakoordeen-Reddy et al 2020	2.1053	[1.6736; 2.6485]	< 0.0001	0.0674	0.2597	46.2%
Omitting Hong et al 2021a	1.9916	[1.6324; 2.4298]	< 0.0001	0.0388	0.1970	42.2%
Omitting Hong et al 2021b	2.1973	[1.7184; 2.8097]	< 0.0001	0.0804	0.2836	46.2%
Omitting Miller et al 2020	2.1791	[1.7576; 2.7016]	< 0.0001	0.0409	0.2022	35.4%
Omitting Reidy et al 2018a	2.1734	[1.7013; 2.7766]	< 0.0001	0.0810	0.2846	46.6%
Omitting Reidy et al 2018b	2.1452	[1.6910; 2.7213]	< 0.0001	0.0754	0.2746	46.6%
Omitting Bell et al 2013	2.0098	[1.6472; 2.4521]	< 0.0001	0.0411	0.2028	41.0%
Omitting Hill et al 2011	2.2163	[1.7381; 2.8260]	< 0.0001	0.0734	0.2709	44.0%
Omitting Raguema et al 2022	2.2055	[1.7222; 2.8242]	< 0.0001	0.0807	0.2841	46.0%
**Random effects model**	**2.0673**	**[1.6799; 2.5439]**	**< 0.0001**	**0.0511**	**0.2261**	**42.2%**
