# Supplementary material for: Preeclampsia Genomic Susceptibility Factors in Populations of African Ancestry: A Systematic Review and Meta-Analysis
Source: Int J Mol Sci. 2026 Mar 12;27(6):2594. doi: 10.3390/ijms27062594 (PMC13027360; doi:10.3390/ijms27062594)
Supplement: Supplementary file 1 [file ijms-27-02594-s001.zip › Supplementary Table S8.pdf]

Supplementary Table S8: Leave one out analysis cellular homeostasis

Omitted study	OR	95% CI	p-value	τ^2	τ	I^2
Omitting Khaliq et al 2020a	1.6631	[1.4244; 1.9417]	< 0.0001	0	0	0%
Omitting Khaliq et al 2020b	1.6367	[1.3966; 1.9181]	< 0.0001	0	0	0%
Omitting Morrison et al 2010a	1.6810	[1.4296; 1.9765]	< 0.0001	0	0	0%
Omitting Morrison et al 2010b	1.6472	[1.3906; 1.9512]	< 0.0001	0	0	0%
Omitting Morrison et al 2010c	1.6411	[1.4034; 1.9190]	< 0.0001	0	0	0%
**Random effects model**	**1.6537**	**[1.4331; 1.9082]**	**< 0.0001**	**0**	**0**	**0%**
