# Supplementary material for: Preeclampsia Genomic Susceptibility Factors in Populations of African Ancestry: A Systematic Review and Meta-Analysis
Source: Int J Mol Sci. 2026 Mar 12;27(6):2594. doi: 10.3390/ijms27062594 (PMC13027360; doi:10.3390/ijms27062594)
Supplement: Supplementary file 1 [file ijms-27-02594-s001.zip › Supplementary Table S9.pdf]

Supplementary Table S9: Leave one out *APOL1* G1/G2

**Omitted study**	**OR**	**95% CI**	**p-value**	**τ^2**	**τ**	**I^2**
Thakoordeen-Reddy et al., 2020	1.6623	1.3545 – 2.0400	<0.0001	<0.0001	0.0010	0%
Hong et al., 2021a	1.6476	1.3488 – 2.0125	<0.0001	0	0	0%
Hong et al., 2021b	1.7478	1.3588 – 2.2482	<0.0001	0.0126	0.1121	5.9%
Miller et al., 2020	1.9228	1.4919 – 2.4782	<0.0001	0	0	0%
Reidy et al., 2018a	1.6855	1.3470 – 2.1090	<0.0001	0.0035	0.0591	3.0%
Reidy et al., 2018b	1.6726	1.3589 – 2.0586	<0.0001	<0.0001	0.0011	1.7%
**Overall random-effects model**	**1.6974**	**1.3950 – 2.0655**	**<0.0001**	**0**	**0**	**0%**
